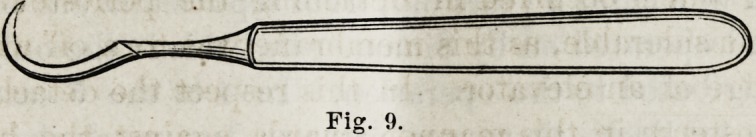# Surgical Treatment of Cleft of the Hard Palate, with an Illustrative Case
*This paper was read at a meeting of the New York Medical Journal Association February 19, 1869.


**Published:** 1869-07

**Authors:** Wm. R. Whitehead

**Affiliations:** New York.


*DR. WHITEHEAD'S CASE OF CLEFT PALATE.*

Before the Operation.

After the Operation.

### SELECTED ARTICLES.

### ARTICLE VI.

### *Surgical Treatment of Cleft of the Hard Palate, with an Illustrative Case.**

By Wm. R. Whitehead, M. D., New York.

Cleft of the hard palate has been regarded by most surgeons as beyond the resources of surgical intervention; and though numerous attempts have been made to establish the operation for this infirmity as a feasible and desirable procedure, yet not until within a few years has this much-desired object been attained.

In 1816, Græfe, of Berlin, was the first to attempt the operation for cleft of the soft palate, but was unsuccessful, and three years later Roux gave brilliant éclat to the operation known as staphylorrhaphy, by the happy success which attended his essay on the person of Dr. Stephenson, who read the report of his own case before the French Academy of Medicine, thus signally attesting the advantages of this triumph of French Surgery. Staphylorraphy then came in vogue with aspiring surgeons: its difficulties were more or less successfully met by ingenious-devised instruments. It had its glories and its defeats, the former being due to the more or less propitious circumstances which attended and succeeded its exact and skilful performance; and in this respect staphylorrhaphy, like some other deservedly well-established operations, has its serious disadvantages. After the first ardor, which its novelty had excited, somewhat abated, numerous objections were urged against it, and this operation declined in the favor of many. The operations for cleft of the hard palate shared a worse fate; and though Roux, Kreimer, J. Mason Warren, Pollock, and some others, met with a certain encouragement, yet the opinions of most medical men regarding operations for the closure of congenital or acquired defects of the hard palate have been strongly marked with

* This paper was read at a meeting of the New York Medical Journal Association February 19, 1869.

dissent; and at the present time the mechanical devices of the dentist are preferred to all operations for cleft palate of every description, by those who are unacquainted with the progress which has been made in this part of surgery. Heretofore there has been just reason to accept gladly any mechanical means, however imperfect, to supply the place of a confessedly tedious and long operation such as staphylorrhaphy. The emulous attempts of surgeons, to close, by operation, defects of the palatine vault, have met with but little encouragement, and this quite independent of a few, and I am happy to say only a few, who like to oppose any innovation which they imagine departs from their fixed and sometimes mistaken convictions, of which they appear more tenacious as they increase in years.

Gentlemen, I ask your indulgent attention to what I have to say about a German operation, and I believe I am the first who has endeavored to introduce it in this country. Many of you, I have no doubt, are familiar with Pollock's successful cases of cleft of the hard palate, and possibly some of you are acquainted with the attempts of Baizeau at restoration of acquired defects of the palatine vault. I shall not detain you with a consideration of the old modes of operating for cleft of the hard palate. It affords me pleasure, however, to remind you that to our honored and lamented countryman, J. Mason Warren, whose signal services in American surgery are respected at home, and widely esteemed abroad, is due one of the most remarkable and singularly successful efforts at closure of an extensive cleft of the hard palate. The direction and extent of the incisions in this case, as subsequently were those used by Pollock, resembled very much those adopted in the German method. But what particularly distinguishes this method is the inclusion of the periosteum within the flaps, with a view to the reproduction of bone. As you are well aware, the subject of the reproduction of bone from the periosteum has been well studied practically in our own city. The comparatively recent publication of the extensive works of Ollier and Sédillot has added fresh and much-increased interest to this suggestive subject. The experiments of Flourens on the periosteum of animals were not lost to science, and some desirable applications to surgery have attested the value of those experiments. Never are we more forcibly reminded of the fortuitous distribution, by the winds, of different seeds falling on barren and fertile places, than in considering the accidental distribution of isolated scientific facts, which often seem of no use until beautifully developed by some fertile intellect. To Langenbeck we are indebted for an eminently useful application of the principles which Flourens had enunciated; to this Prussian surgeon is due a very successsful operation which, while it includes the periosteum in the flaps, with a view to the ultimate reproduction of bone to close the fissured vault, preserves the nutrient vessels uninjured, and thus contributes to success, by the avoidance of gangrene and sloughing of the flaps.

Thus far I have had but a very limited experience with this desirable operation, but I shall request presently to show you one of my cases which was recently operated on, and beg to call your attention to the one reported in the July number of the *American Journal of the Medical Sciences* for 1868, and which some of you saw at the meeting of this Association about a year ago, but before Langenbeck's method was essayed. I regret that the lady manifests some reluctance to appear here this evening, otherwise you would have an opportunity of seeing the very satisfactory result which has been obtained.

There is another case which has been operated on by me, an acquired defect of the hard palate, and which appeared to be a most favorable case for operation. In this case I signally failed, and I exhibit to you a plaster cast of it taken by a dentist for me before the operation. My failure was due principally to an exceedingly profuse suppuration; though I shall not attempt to exonerate myself entirely from the neglect to observe one or two little precautions in the performance of the operation, and which I have since learned to value more highly. Also I am more thoroughly impressed with the importance of an entire eradication of the constitutional disease which sometimes causes the defect, before attempting an operation. In a case which came under my care a few days ago, caused by syphilis, I expect to assure myself as well as possible, by months of attentive observation, of the elimination of all constitutional taint before I operate. I propose this evening to give you only a general description of this new method of operating for cleft of the hard palate, and for more ample details I beg to refer you to an extended paper by me on this subject, in the October number of the *American Journal* for 1868. I shall not presume to repeat here verbatim that which is quite accessible to all of you who may desire to consult this article at your convenience; but I shall request permission to refer if necessary, to the woodcuts contained in it. I think that there can be fairly claimed for this operation advantages which entitle it to a prominent place in the list of useful operations. While I am quite sensible to the objections, some of which may with reason be urged against it, yet the undeniable advantages in its favor may justly claim your attention. In giving a brief history of the case which I offer for your inspection this evening, I shall endeavor to embody in it the most important points necessary to a comprehension of this operation, designated by the somewhat dissonant term, muco-periosteal uranoplasty. But previously I request your attention to the peculiar distribution of the arteries which supply the roof of the mouth. The descending or superior palatine artery, as you know, before emerging from the palatine canal, gives off a few small branches, which pass down the small accessory palatine canals, and are distributed to the muscles of the soft palate and mucous membrane. The superior palatine, in its horizontal portion, runs along in a groove at the junction of the horizontal plates of the maxilla and palate-bones with the alveolar process (see Fig. 1). Anteriorly

Fig. 1.

this artery passes through the anterior palatine canal upward to anastomose with the one on the opposite side and the artery of the septum nasi. The horizontal portion of the superior palatine is of considerable size, and is included in the periosteal flaps when they are detached from the bone. But these flaps remain adherent at their three nutrient points which correspond to the orifices of the spheno-palatine canals and the anterior palatine canal. The superior palatine is of considerable size, and if cut may occasion troublesome hæmorrhage. But this accident can be readily avoided by carefully detaching the periosteum with a blunt periosteal elevator. There is a little branch of the ascending palatine which, after passing between the tendons of the levator and tensor-palati muscles, is in relation with the inner border and posterior surface of the tensor-palati muscle, and the knife, in dividing this muscle during the operation to relax the velum palati, cuts this little branch, and nearly always causes some bleeding, but which, however, can be conveniently checked with ice-water spray thrown on the part.

There are numerous differences in the form, extent, and general appearance of cleft palates. They are very naturally divided into those which are congenital, and those which are the consequence of disease, as syphilis or scrofula, or of accident, the result of gunshot wounds of the mouth. After operations on the palatine vault, as in the removal of tumors of this region, or after resection of the upper jaw, the surgeon may be called upon to close, by operation, defects of the hard palate. The congenital defects are more or less familiar to you all. Most usually, the cleft does not extend beyond the middle of the horizontal plates of the maxillæ. Very often the fissure is complete, and there is separation of the alveolar process in front; sometimes with a disfiguring projection of the intermaxillary bones. Occasionally there is a double cleft of the hard palate, complicated or not with double or single hare-lip. Exceptionally, there is seen a congenital defect of the hard palate only, and the soft palate is not split. Usually, however, whenever there is cleft of the former, there is complete separation of the velum. The cleft is quite often median—that is, occupying the middle of the vault; but, very frequently, it is more to the left. There are infinite varieties and degrees of this infirmity; but the operation, as applied to one of the not infrequent forms of cleft of the hard palate—such as that to which I now ask your attention—will suffice to illustrate the main features of the operation. But, on account of the very incomplete development of the lateral halves of the cleft velum in this case, the improvement in speech will necessarily be less marked than it would be were that development more considerable. But I do not wish to anticipate now what I have to say to you concerning the influence of this operation on the speech; and this is a point which should, and I feel confident will, engage your attention, as being one of the most attractive features of this German method.

Case.—Maria D., æt. 7, had a complete cleft of both the hard and soft palate. The cleft originally extended through the alveolar process in front, and was complicated with hare-lip, which last had been operated upon before I saw her, leaving an ugly-looking notch, which, however, can be readily closed. The cleft was five eighths of an inch posteriorly, and gradually diminished toward the front at the alveolar process (see plate). The deformity, as it appeared before the operation, is well exhibited by a plaster cast of the roof of the mouth, taken by a dentist previous to the operation, and which I offer for your examination.

On the 16th of last December, I closed the whole of the cleft by suture, most efficiently assisted by Drs. Louis Elsberg, F. A. Burral, Octavius White, and Dr. Robert Newman. This last gentleman kindly administered ether. The operation was long and tedious, and required considerable patience.

Fig. 2.

This stout wire gag (see Fig. 2), which I hold in my hand, was used to keep the mouth open during the operation. I think that this instrument is very well suited to many operations on the mouth, and has been much improved by the addition of a tongue depresser. The patient was placed on a sofa opposite a window, but the light was so dim that afterward artificial illumination was used.

After the administration of ether, the palato-pharyngeus, palato-glossus, and levator-palati muscles were severed, and the operation continued; but not until after the loss of considerable time from vomiting by the patient. There was also some delay occasioned in arresting the bleeding, and in washing out the throat with a spray apparatus.

The point of a sharp knife, curved on its surface (See

Fig. 3.

Fig. 3), was passed around and behind the hamular process, and over the lower part of the internal pterygoid plate, so as to cut loose the mucous membrane which confined each lateral half of the velum to this part.

If there had been a very perceptible ledge of bone, formed by the horizontal process of the palate-bone, it would have been necessary, as Langenbeck recommends, to detach the mucous membrane from the posterior border of this vestige of the palate-bone; and this is a very important precaution, as otherwise the flaps will not fall together as they should after other stages of the operation are completed. This was the most difficult and tedious part of the operation, and is generally so considered. An incision was next made along the edges of the cleft. It is proper here to remark that, if there be a ledge of bone, this incision should run along its edge, being careful, in every case, to keep at least an eighth of an inch beyond the groove in which courses the superior palatine artery. This incision should be made to the bone, and run along on its surface.

Fig. 4.

Other cuts were next made, one on each side, which extended from about the eye-teeth to slightly beyond the last molars, and along the border of the gum (see A and B, Fig.

4, copied from Langenbeck). These cuts were made through the periosteum and to the bone.

Such extensive cuts may not always be necessary, and *interrupted side-cuts* have sometimes been preferred. For detaching the periosteum, I made use of an instrument like this (see Fig. 5)—

Fig. 5.

which Dr. Sayre facetiously calls his oyster-knife, and which he uses most advantgeously in detaching the periosteum in operations on the hip-joint.

Gentlemen, this instrument is far superior for this purpose to any periosteal elevator that I have seen, and much more handily used than that of Langenbeck, which I here exhibit:

Fig. 6.

In detaching the periostoum, the nutrient parts of the flaps, to which I have alluded, were carefully respected ; and after this stage of the operation was completed, the flaps almost met in the middle line. The paring of the edges was next done, and the passage of the sutures, seven or eight in number, was readily accomplished by means of this suture-needle—

Fig. 7.

which I claim to be better than that used by Langenbeck or others in this operation. A silver-wire canulated needle was essayed in this case for making one suture as on a previous occasion; but I prefer the needle which I have shown you as being more simple.

Some of the wires were twisted, and some of them were tied, as Sims's instrument, which I had made use of for adjusting the wires, had become misplaced, and could not be found until after the operation.

Bits of cotton were stuffed in the side-cuts, to keep them from healing too soon, and, also, by the pressure which they exerted on the sides of the flaps, to extend the line of union.

The hæmorrhage was quite abundant for a few minutes, but was readily controlled by means of ice-water spray. This bleeding came from the little branch of the ascending palatine artery, when I divided the levator-palati muscle. The bleeding which occurred in detaching the periosteum was very inconsiderable, as this membrane was torn off with the blunt edge of an elevator. In this respect the detaching of the periosteum in this manner guards against the hæmorrhage, which was formerly the accompaniment of *mucous* uranoplasty. On one occasion, my first uranoplastic operation, I cut the superior palatine artery, but controlled the hæmorrhage, without much trouble, by compression and the use of styptics.

Langenbeck states that, "in 14 cases in which mucous uranoplasty was done, there were six cases of dangerous secondary hæmorrhage; whereas, in 25 cases in which the periosteum was detached, there was no after-bleeding."

The patient was put to bed, and some strong beef-tea and other liquid food, as milk, ordered, as her only nourishment for ten days. There was no sloguhing or profuse suppuration following this operation. Spray containing carbolic acid was thrown twice a day into the side-cuts, into the throat,and through each nostril into the nose; great care was taken not to project the spray on the line of union. The cotton plugs were removed several times, and fresh ones saturated with glycerine put in their places. In the left side-cut some of the cotton remained much longer than I intended, and was not discovered until all the sutures were removed. Most of the sutures were taken out about the 14th day, and the last ones remained until the 18th. The union was found complete all the way through (see plate): and the parts at present feels as if solid bone will ultimately be formed. This point however, I prefer to test a little later with a needle.

For making the side cuts, I used a knife like this:

Fig. 8.

and for dividing the pillars of the fauces a sickle-shaped knife like this:

Fig. 9.

There is a brief analysis of 55 cases compiled by me and published in the *American Journal.* This analysis I have since reduced to a tabular form, which is prepared with some care, but I will not tax your attention by reading that which may seem unnecessary.

I shall content myself, therefore, with a short recapitulation of the most salient points of this analysis, and especially allude to those which I am sure will appear of most interest. Of these 55 cases collated by me, though they are far from representing the actual number of cases operated on, 47 of them were congenital defects. The remaining 8 cases were acquired deficiencies of the hard palate. Most of these operations were performed by Langenbeck and by Simon. I shall allude first to the cases of congenital cleft palate. Of these there were, of the 47 cases mentioned, 35 in which there was in each a complete cleft of both hard and soft palate. In 3 of these there was a double hare-lip—in 2 a single hare-lip—and in 5 a double cleft of the vault, one of which five cases was complicated with a double hare-lip.

There were 10 cases in which there was an incomplete cleft of the hard, and a complete cleft of the soft palate. In one case there was no mention made of the nature of the defect, and the remaining case was one of partial cleft of the hard palate without separation of the soft palate.

At first, and in some of the cases operated by Langenbeck, staphylorrhaphy was performed before uranoplasty; in fact, in 13 of the 47 case, staphylorrhaphy preceded uranoplasty. in 5, uranoplasty was done first, and in 24 cases uranoplasty and staphylorrhaphy were performed at one operation. In 5 cases uranoplasty alone was done. Silver sutures were used in only a few of the cases.

There were 33 cases of complete cure—9 cases in which the cure was incomplete, and 5 complete failures, one of them a death in a child 2 years old, from septicæmia on the 10th day, and operated on by Simon. But this is the only death ever attributed to the operation. In 4 of the 5 cases operated on by Beck, there was more or less marked improvement in speech, and in his 5th case, which was a very incomplete cure, we are led to infer that there was none. In two of his cases, he speaks of the nasal tone persisting. The three cases operated on by Billroth were on children. Two of these cases were failures, one of them an infant eight weeks old. The case which succeeded was of a child 28 weeks old; but nothing is said of the subsequent effects of the operation on the speech. Of 21 cases by Langenbeck, of which I have made an abstract, one was a failure, and there were 4 cases in which the cure was incomplete. Of the 16 cases out of 21 in which the cure is reported by him as complete, or nearly so, there are 5 cases in which he does not allude to the speech; in one of them, a woman 22 years of age, there was a good formation of bone. In another, a lad 9 years old, there was a very fine form of the vault obtained, but no new osseous formation. The three remaining of these five cases were youths of the ages of 9, 15, and 18. In 11 out of these 16 cases cured by Langenbeck, there was more or less marked improvement in speech. In some, the speech, which was unintelligible before the operation, became intelligible and continued to improve. In some the improvement in speech was only slight, in others the speech became perfectly distinct. In a single case operated on in France, by Sédillot, on a child 3 years of age, there is nothing said about the speech; but the only object of this operator seems to have been to disprove that the periosteum alone can in this locality or elsewhere reproduce bone. In the 15 cases of congenital cleft of both the hard and soft palate operated on by Simon, the speech in 4 of them is reported by him as nasal and hard to understand. In 6 of the remaining 11 cases the speech was improved and made intelligible, but it remained more or less nasal. In the other 5 cases there were two of them in which uranoplasty alone was done, and the speech was not improved; in the other 3 cases either the hard or soft palate reopened or failed to unite, and the speech was about ths same as before the operation. In the case of my patient mentioned in the analysis, the speech, which before the operation was unintelligible, afterward became distinct and intelligible, but the nasal tone still persists.

We come now to acquired defects of the hard palate, the result of disease or wounds; and, of the 8 cases cited in my analysis, nearly all of them were attended with immediate restoration of normal speech. In order to examine thoroughly the advantages and disadvantages of this operation on the speech, it is proper to consider the influence of the velum palati on the modulation of the voice. Simon has most thoroughly studied this subject, and critically examined it. The persistence of the nasal tone, to which he has pointedly called attention, would seem somewhat to impair the usefulness of the operation when applied to cleft of the hard palate, involving a separation of the velum. There can be no doubt, that when the cleft is very considerable, and the two halves of the bifid velum are exceedingly small and undeveloped, there is not enough tisssue out of which to construct a movable and serviceable soft palate, which shall even moderately well supply the offices of a normally-developed velum.

The movements of the velum during speech cause the buccal and nasal cavities to communicate, or to be occluded from each other. When a complete occlusion cannot be effected, as in cleft palate, or from any other cause, such as the paralysis of the palatine muscles, temporarily resulting from diphtheria, more or less nasal tone must be the consequence. It is necessary, however, that a certain degree of nasal tone should occur in the pronunciation of certain words, in order to constitute purity of speech. If a nasal tone accompanies the pronunciation of nearly every word, as is sometimes even strikingly observed in persons who are not affected with cleft palate, the speech may be much marred; and, should it be characterized by an indistinct, harsh, and guttural articulation, I can conceive of nothing more displeasing to a person whose hearing has been cultivated to harmonious sounds. We know, however, that some persons who have a marked nasal accent have, nevertheless, a remarkably clear and distinct articulation. This is more perceptible in the French language than in our own.

Whenever cleft of the hard palate is accompanied with a wide separation of the velum, and when this last is not much developed, distinctness of speech may be obtained by the combined operations of uranoplasty and staphylorrhaphy, but the nasal tone will always persist, because the newly-formed palate will be too short, and the united velum too tense to permit at will the occlusion of the nasal from the buccal cavities. But distinctness of speech is a most desirable object to a person with cleft palate, whose articulation is unintelligible. Moreover, the most skillfully-devised mechanisms, of which I know none better than that of Kingsley, will not replace the permanent benefit which the operation of periosteal uranoplasty may afford to cleft of the hard palate.

Though this operation may be somewhat irksome to both the operator and patient, it is attended with very little danger to the latter.

The flowing downward into the throat and stomach of the nasal secretions is prevented afterward ; and this is no inconsiderable advantage to those who are inflicted with such a repulsive infirmity as an extensive cleft of the hard palate. An obturator, with a soft-rubber velum properly adjusted to it, may admit of temporary advantages, but like some other mechanisms, it is only a make-shift, and is liable to become disarranged. It needs repairs, and requires frequent cleansing.

Obturators, in order to fit accurately, must be adjusted to the increasing size of each young patient's mouth, and every six months a new obturator may be needed. They are often sources of irritation ; they wear the teeth to which they are sometimes attached, and frequently oppose the spontaneous closure or lessening of small perforations of the palate. These devices may be swallowed ; in fact, in one instance death was the consequence of asphyxia from this cause. They are in some respects much like some of the artificial limbs which promise much and are at first very attractive both to the patient and to the surgeon ; but these artistic wooden legs are after a while thrown aside to give place to crutches, or are availed of only on grand occasions, being designed more for show than for use.

Obturators, however, have their uses, and may sometimes advantageously replace the operation of muco-periosteal uranoplasty.—*New York Medical Journal.*

---

## Figures and Tables

**Figure f1:**
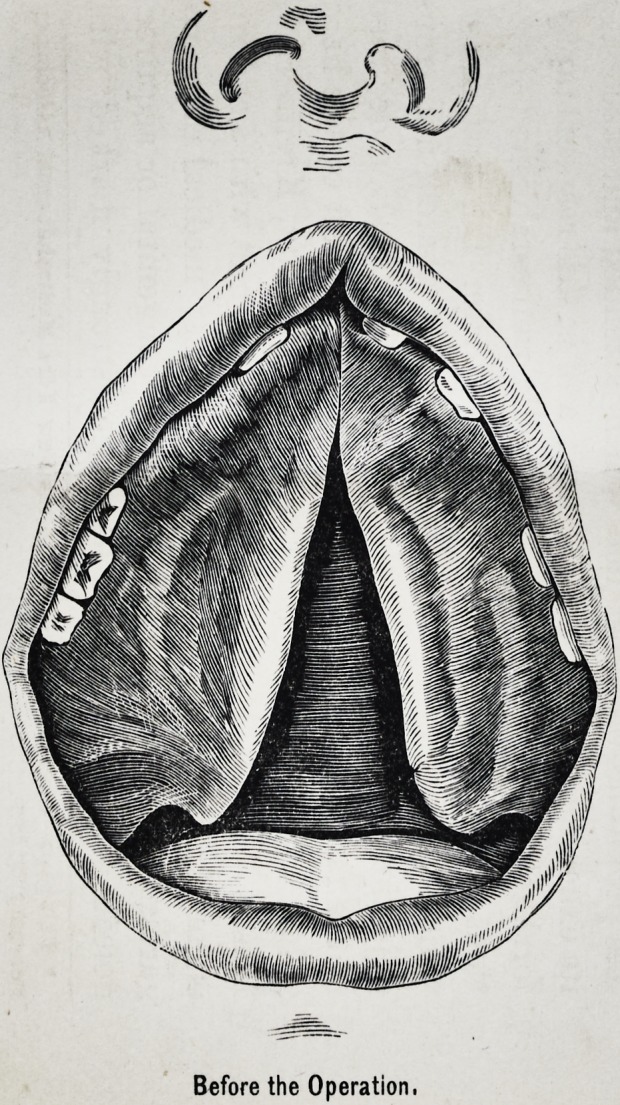


**Figure f2:**
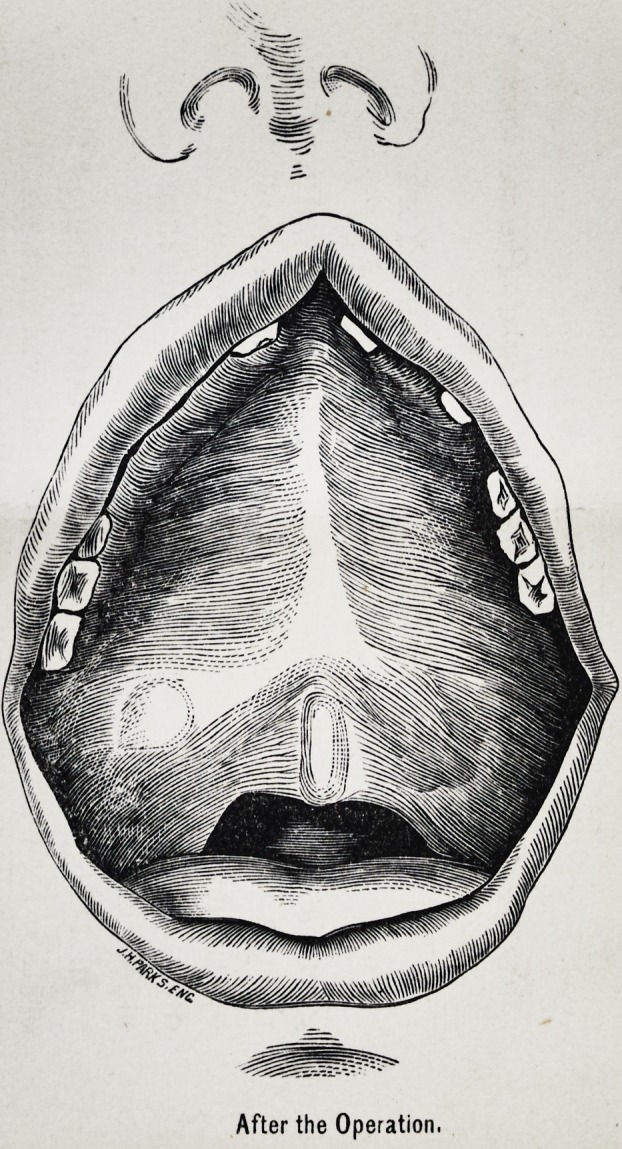


**Fig. 1. f3:**
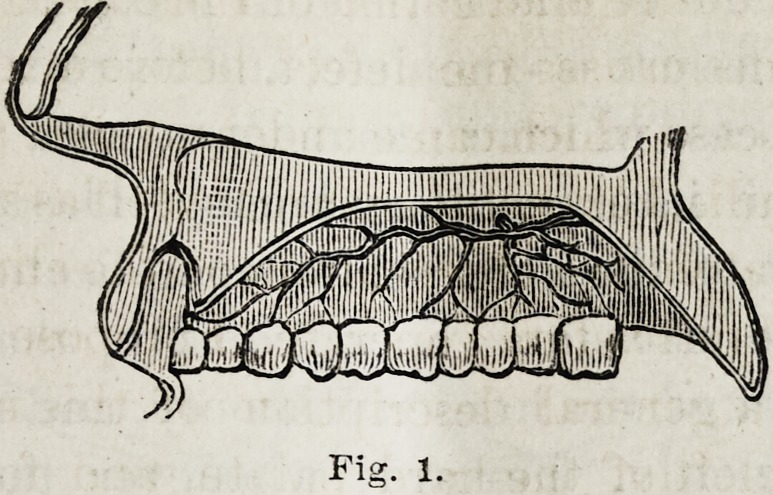


**Fig, 2. f4:**
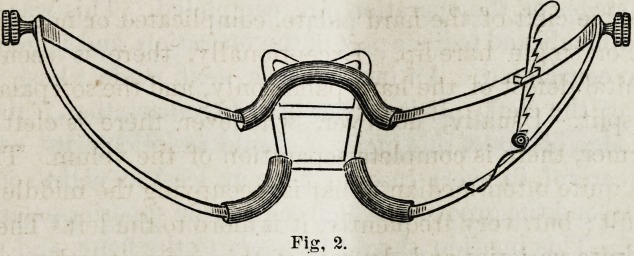


**Fig. 3. f5:**
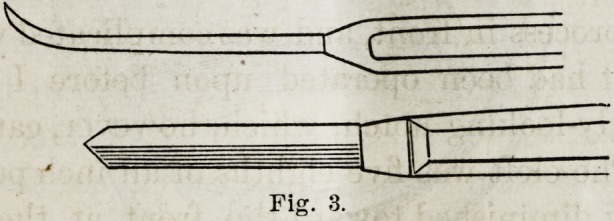


**Fig. 4. f6:**
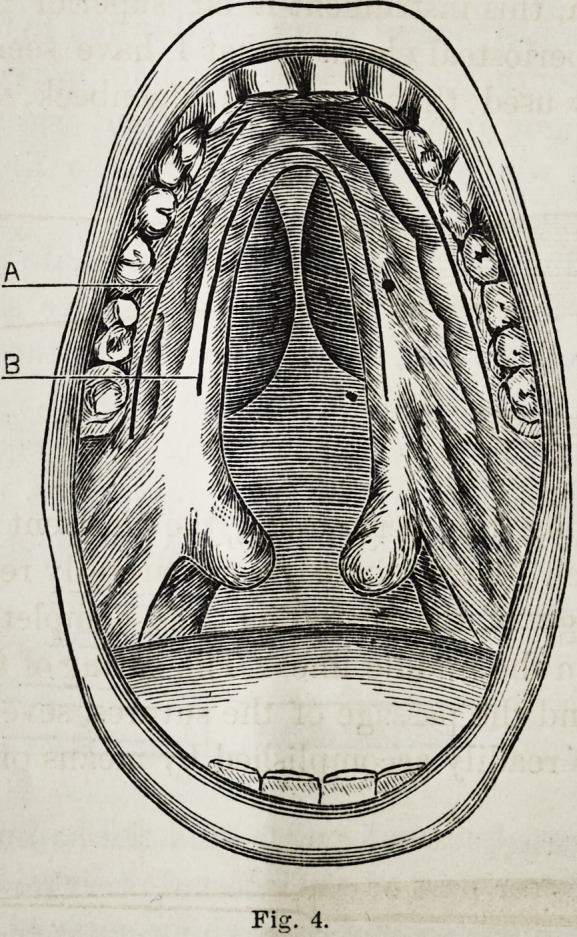


**Fig. 5. f7:**
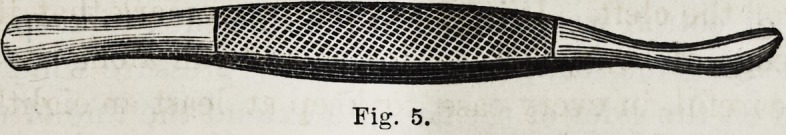


**Fig. 6. f8:**
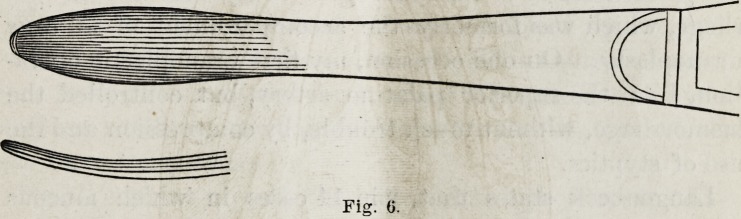


**Fig. 7. f9:**
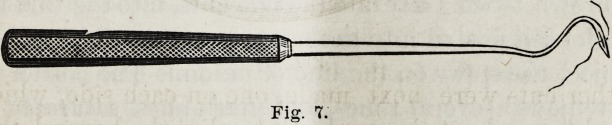


**Fig. 8. f10:**
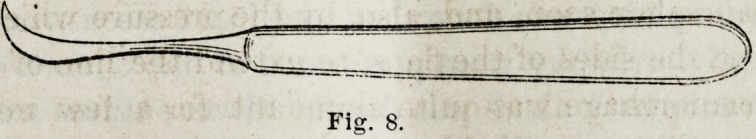


**Fig. 9. f11:**